# From Happiness Orientations to Work Performance: The Mediating Role of Hedonic and Eudaimonic Experiences

**DOI:** 10.3390/ijerph16245002

**Published:** 2019-12-09

**Authors:** José M. Peiró, Malgorzata W. Kozusznik, Aida Soriano

**Affiliations:** 1 IDOCAL, University of Valencia, 46010 Valencia, Spain; IVIE, 46020 Valencia, Spain; 2 Work, Organizational and Personnel Psychology Research Group, KU Leuven, 3000 Leuven, Belgium; gosia.kozusznik@kuleuven.be; 3 IDOCAL, University of Valencia, 46010 Valencia, Spain

**Keywords:** orientations to happiness, well-being, performance, hedonic, eudaimonic

## Abstract

In organizations, psychologists have often tried to promote employees’ well-being and performance, and this can be achieved through different pathways. The happy-productive worker thesis states that ‘happy’ workers perform better than ‘unhappy’ ones. However, most studies have focused on hedonic well-being at the expense of the person’s eudaimonic experience. This study examines whether orientations to happiness (i.e., life of pleasure/meaning) are related to hedonic (i.e., perception of comfort) and eudaimonic (i.e., activity worthwhileness) experiences that, in turn, improve performance. We applied multilevel structural equation modeling to diary data (68 office workers; *n* = 471 timepoints). We obtained significant effects of: life of pleasure on self-rated performance through activity worthwhileness, life of meaning on performance (self-rated, rated by the supervisor) through activity worthwhileness, and life of meaning on performance rated by the supervisor through perception of comfort. Results show more significant paths from/or through eudaimonia to performance than from/or through hedonia. The results suggest that the pursuit and/or experience of eudaimonic happiness is more beneficial for work performance than the pursuit and/or experience of hedonic happiness. Theoretical and practical implications for organizations are discussed.

## 1. Introduction

There can be different pathways to achieving better performance at work; however, those that involve meaningfulness may be among the most effective [1,2,3,4]. The promotion of well-being and performance at work is one of the main interests of the organizational health framework [5]. In this regard, the happy-productive worker thesis [6], which states that ‘happy’ workers perform better than ‘unhappy’ ones [7], has been established as a referential model when studying these two work outcomes. However, the literature reveals that studies that have analyzed the relationship between well-being and performance have a limitation, which is their excessive focus on hedonic well-being (e.g., job satisfaction or job-related affective well-being) at the expense of the eudaimonic experience (e.g., meaning at work or purpose in life) [8]. Indeed, well-being can be pursued through two distinct but complementary ways: the hedonic pursuit of pleasure and the eudaimonic pursuit of meaning [9].

The issues of whether pursuing pleasure or purpose actually leads to having more hedonic or eudaimonic experiences [10], and whether these experiences improve work performance [11], have been identified as important questions for research, although they have not been sufficiently studied. The orientations to happiness (OTH) theory [10] proposes that well-being can be pursued through two distinct but complementary ways: the hedonic pursuit of pleasure and the eudaimonic pursuit of meaning [9]. These two variables are independent, but not mutually exclusive, and together they offer a complete picture of well-being [12]. Moreover, as postulated by the affective events theory (AET) [13], such trait-like personal orientations are expected to influence momentary affective experiences, in this way, affecting performance. In other words, stable personal characteristics can shape performance only when specific experiences take place. In turn, the OTH theory provides an additional lens to explain these relationships and clarifies that individuals with a hedonic orientation will experience hedonic well-being (e.g., perception of comfort) as more salient, whereas individuals with a eudaimonic orientation will experience eudaimonic well-being (e.g., activity worthwhileness) as more salient [14]. That is why the combination of both OTH and AET theories can help to explain performance in a more efficient way than each of these two theories separately.

These theoretical proposals have been corroborated by recent studies. For example, Reference [15] showed that each OTH domain influenced its matching momentary experience domain, which means that a life of pleasure influenced the experience of momentary pleasure, and a life of meaning influenced the experience of momentary meaning. In addition, other research shows that each OTH domain can influence its non-matching momentary domain; thus, a relationship has been found between a life of pleasure and the experience of momentary meaning, and between a life of meaning and the experience of momentary pleasure [16,17].

Simultaneously, some studies showed a positive relationship between daily experiences of well-being, such as perception of comfort [18,19] or activity worthwhileness [20], and work performance. However, the research has predominantly focused on the hedonic aspect of well-being, and little is known about how eudaimonic well-being might affect performance-related processes [11].

Consistent with the predictions of AET and the empirical evidence on the existence of a mediation chain from affective dispositions to work performance through affective reactions [21,22], in the present study, we aim to expand this mediation chain to include the mediating role of *both* hedonic and eudaimonic daily experiences in the relationship between OTH and work performance. With this in mind, the purpose of the present research is to study whether different types of OTH (i.e., life of pleasure and life of meaning) are related to hedonic (i.e., perception of comfort) and eudaimonic (i.e., activity worthwhileness) daily experiences, which, in turn, would improve work performance (self-reported by the employee and rated by the supervisor). The present study contributes to finding the “best paths” through which an individual’s orientation to happiness may lead to improved organizational behavior.

### 1.1. What are Orientations to Happiness?

Pursuing optimal well-being is an endless struggle in human beings [23]. In this regard, the OTH framework [10] follows the philosophers and psychologists who have long been concerned with the good life and how it can be achieved [24,25,26]. This model deconstructs happiness into different components or orientations [27]. According to the OTH framework, people can seek happiness in (different aspects of) life in two distinct ways that are not incompatible and may be pursued simultaneously [10]: the hedonic pursuit of pleasure and the eudaimonic pursuit of meaning [9]. The hedonic perspective considers that well-being can be attained through the pursuit of enjoyment, pleasure, and comfort [12]: “Don’t worry—be happy” [10]. In turn, the eudaimonic perspective assumes that well-being can be achieved through a full application and development of oneself [28,29]: “Be all that you can be,” and “Make a difference” [10]. The OTH framework is an example suggesting that people may reliably differ in the type of life they pursue [30], although they might also orient their pursuits toward both perspectives of well-being at the same time [10].

On their orientations to happiness scale, Peterson and his colleagues [10] assess individuals’ pursuit of well-being corresponding to both hedonic and eudaimonic perspectives. The life of pleasure subscale is considered as an index of hedonic pursuit, and it refers to individuals’ attempts to amplify the intensity and duration of the experience of various positive emotions [31]. In turn, the life of meaning subscale is considered as an indicator of the eudaimonic pursuit, which is defined by the authors as persons using their “skills and talents in the service of greater goods—including, in particular, the welfare of other people or humankind” [10]. The orientations to happiness scale contains an additional subscale (i.e., life of engagement) in Peterson and his colleagues’ [10] approach. This subscale aims at measuring flow, although there is no agreement in the literature about whether it is part of eudaimonic well-being [32] or a third orientation to happiness [10]. Therefore, following the researchers who point to the inconsistent arguments about this construct [17] and other researchers who are interested in the study of the hedonic and eudaimonic perspectives [33], in the present research, we focus on the life of pleasure and the life of meaning orientations to happiness.

### 1.2. The Impact of the Orientation to Happiness on Well-Being Experiences

Hedonic and eudaimonic OTH can each be associated with different outcomes [10], and we can “assume that given orientations shape conduct” ([10] p. 37). Indeed, they are considered traits that can determine which activities different people will pursue [15]. Accordingly, following the AET [13], individual trait differences (such as in OTH) are expected to influence momentary experiences. For example, the AET states that people high in traits such as dispositional positive affectivity might be especially responsive to potentially pleasing events in the work environment [34]. In the same vein, stable characteristics such as distinctive and pervasive positive affect can “give rise to both intra-individual variations in emotional state and inter-individual differences in emotionality” [35]. Therefore, based on the AET, we should expect traits such as the hedonic orientation to happiness to lead people to engage in and be especially responsive to potentially pleasing situations at work, like the state of physical comfort (often operationalized as perception of lack of environmental stressors [18,36]), whereas the trait orientation to a life of meaning should lead individuals to engage in and be especially responsive to work activities that convey meaning and can be described as being especially worthwhile. Hence, the OTH framework assumes that people who believe in pursuing pleasure have more sensually gratifying experiences than those who do not [10], and that people who believe in pursuing meaning are more likely to apply their personal strengths [27].

Despite the interest in the relationship between OTH and well-being experiences, few studies have looked at self-reports during or just after actual behavior [15], employing, for example, a diary design or experience sampling methods (ESM). This might be because, as noted by several authors [30,37], the OTH questionnaire obtains self-perceptions of behaviors, rather than information about what people actually do [15]. Given that there could be discrepancies between endorsement (OTH) and the actual experience, the extent to which endorsement predicts or does not predict the actual experience requires further investigation [16]. Furthermore, an important issue proposed for future research is whether people who say they believe in the pursuit of pleasure actually have more sensually gratifying experiences than those who do not, and whether people who agree with statements that refer to a life of meaning are more likely to perform service to others [10]. However, the research on this topic is scarce. A rare exception is the research by Grimm, Kemp, and Jose [15], who used ESM to show that trait orientation to happiness can influence the pursuit and experience of everyday activities. Specifically, they found that an orientation to pleasure was a positive significant predictor of experiences of pleasure, and an orientation to meaning was a positive significant predictor of experiences of meaning. Moreover, Henderson, Knight, and Richardson [17], using the day reconstruction method (DRM), found that the orientation to a meaningful life predicted higher eudaimonic behavior. This issue was also partially addressed by Waterman [29], who asked respondents to report on “activities of importance” and their features, although he did not actually measure daily experiences. In light of the existing research in this area, we consider it necessary to use diary methods that would allow us to capture actual hedonic and eudaimonic experiences at work (i.e., perception of comfort and activity worthwhileness) during or just before the actual behavior of employees who present different OTH.

It is important to highlight that the relationship between OTH and hedonic/eudaimonic experiences is not straightforward. Specifically, Grimm and colleagues [15] showed that each of the OTH domains also shared a relationship with its non-matching momentary domain. For example, a life of meaning significantly predicted not only the experience of momentary meaning, but also the experience of momentary pleasure. In the same vein, a life of pleasure was a significant positive predictor of the experience of momentary meaning. Additionally, in his research with a sample of teachers, Chan [16] showed that having a greater meaningful life orientation was a significant predictor of experiencing more positive affect and satisfaction. Taking this research into account, the existence of cross-relationships between orientations to happiness and their non-matching momentary domains should also be considered. Although the research these authors propose makes an important contribution, they do not frame their results in a broader organizational context because they do not evaluate the possible impact of the OTH and subsequent daily experiences at work on organizational outcomes (e.g., work performance).

### 1.3. The Impact of Well-Being Experiences on Work Performance

Performance is usually defined as including task- and context-related performance [38]. Task performance is related to task fulfillment, that is, the quality of task performance refers to the degree that a person fulfills the requirements of his/her job. Context-related performance [38] and organizational citizenship behavior [39] refer to the employee who goes beyond his/her immediate job duties in order to contribute to the well-being of workfellows and the functioning of the whole organization by offering help to his/her colleagues, and investing time and dealing with problems even when they are beyond his/her immediate responsibility specified in his/her job description [19].

According to the happy-productive worker thesis, a happy worker has a greater probability of being a productive worker [40,41]. Indeed, some research shows that well-being can predict performance [11]. For example, when people are more satisfied with their jobs, they show greater performance [42,43] and productivity [44]. Simultaneously, according to Huta and Ryan [13], AET momentary affect may influence momentary behaviors in real time, referred to as ‘affect-driven behaviors.’ Indeed, positive affect has been shown to predict performance quality [45,46].

#### 1.3.1. The Impact of Comfort Experiences on Work Performance

Hedonic well-being originates from the works of Aristippus of Cyrene (435–356 B.C.), and it refers to contents that involve experiences of pleasure and comfort [14]. In turn, physical comfort can be defined by the absence of individual perceptions of unpleasant factors [18] or stressors that have traditionally been linked to environmental aspects such as lighting, ventilation, and temperature [36].

There has been great interest in the way hedonic well-being might affect performance-related processes [11]. Most studies on well-being and its consequences have adopted a cross-sectional approach [47], investigating its general tendencies and understanding well-being as global evaluations of satisfaction (e.g., job satisfaction). However, given that well-being at work is a dynamic phenomenon, a dynamic research approach (e.g., diary design) is needed to capture the nature and impact of its states (e.g., comfort at work).

Interest in the way the work environment affects employees has grown in recent decades in organizational psychology, with mounting evidence that the workspace affects the way people perform [36]. In addition, there is a widespread belief that a causality exists between excellent or poor indoor environmental quality and productivity gains or losses, respectively [48]. Environmental psychology theory suggests that people’s environment has an impact on their behavior [49]. In fact, different aspects of the indoor environment have been studied, showing that about 86% of productivity problems reside in the indoor work environment [18]. However, as Kozusznik and colleagues [50] found, the perception of employees’ working environment (i.e., environmental stressors) is the key to work outcomes. Indeed, as another study shows, both task- and context-related performance are affected by employees’ level of comfort, which means that environmental comfort can be a very influential aspect in employee performance [18].

Comfort (i.e., lack of perception of environmental stressors) in offices can impact in-role and extra-role performance in several ways. To begin with, people who feel better than usual at work have been found to invest more effort in their tasks [51,52] and achieve a higher level of task performance [53]. Along these lines, Wargocki and colleagues [54] showed that when subjects perceived air quality to be unsatisfactory, there were significantly lower levels of reported effort during text typing and calculation tasks, compared to when the subjects perceived air quality to be satisfactory. Moreover, Lan and colleagues [55] showed that discomfort provoked by elevated temperatures has a negative effect on performance in tasks such as text typing, addition, neurobehavioral tests, Stroop, and choice reaction time. Similarly, improved air quality has been shown to be associated with increased performance in simulated office work (i.e., text typing, addition, and proof-reading) [56]. In the same vein, performance has been found to decrease in the presence of stressors, such as excessively cool or warm environmental temperatures [55], bad indoor air quality [57], or noisy offices, with the latter reducing employee productivity by as much as 40% [58]. Finally, workers in open-plan workspaces tend to perceive noise as a stressor that causes lower productivity [59].

Simultaneously, although some research shows evidence for decreased task performance in the presence of stress [60], people are quite capable of maintaining task performance under stress for a long time [19]. Nonetheless, this situation can have costs in terms of contextual performance, such as a decrease in organizational citizenship behaviors [19]. In fact, the presence of acute stressors has been associated with a reduced tendency to offer assistance to others [61], social withdrawal [19], or an increase in conflict due to behaving in an irritated (and irritating) way [62]. Therefore, the implication might be that people under (environmental) stress are less likely to contribute to the well-being of the community as a whole, which is a key aspect of citizenship behavior [39]. However, on the positive side, Fisher [21] shows that experiences of hedonic well-being are positively associated with helping behavior (i.e., extra-role performance). With this in mind, we formulate the following hypotheses:

**Hypothesis** **1.** *The relationship between a life of pleasure and work performance (self-rated and rated by the supervisor) will be mediated by the perception of comfort.*

**Hypothesis** **2.** *The relationship between a life of meaning and work performance (self-rated and rated by the supervisor) will be mediated by the perception of comfort.*

#### 1.3.2. The Impact of Activity Worthwhileness on Performance

Although most of the research has studied the relationship between well-being and work performance from the hedonic perspective, it only tells half the story because well-being can also be understood from the eudaimonic perspective as the experience of meaning at work [63] over time [64]. This is reflected in the currently available measures of subjective well-being [65], which distinguish between hedonic experiences at work and the ‘worthwhileness’ associated with the activities people carry out while working [66,67,68]. However, researchers acknowledge that little is known about how eudaimonic well-being might affect performance-related processes on a daily basis [11]. In this area, as in the case of hedonic well-being, the research has predominantly focused on general individual dispositions or overall reports of purpose in life (or job), rather than day-to-day experiences of ‘worthwhileness’, which are possible to capture using ESM or a diary research design.

Although the research on the association between eudaimonic well-being and performance is scarce [11], there is some empirical evidence for this association. For example, Niessen, Sonnentag, and Sach [20] demonstrated that, on days when employees perceived increased meaning at work, they also reported being more focused on tasks and behaving in a more exploratory way (i.e., carrying out more information searches), compared to days when they perceived that the work they were doing had less meaning for them. Additionally, research indicates that eudaimonic well-being is also positively linked to contextual performance, as “worthwhileness” has been associated with positive outcomes in terms of organizational citizenship behavior [69]. However, there is a lack of research on the role of eudaimonic well-being in performance-related processes [11]. Taking it all into account, we formulate the following hypotheses:

**Hypothesis** **3.** *The relationship between a life of meaning and work performance (self-rated and rated by the supervisor) will be mediated by activity worthwhileness.*

**Hypothesis** **4.** *The relationship between a life of pleasure and work performance (self-rated and rated by the supervisor) will be mediated by activity worthwhileness.*

**Figure 1:**
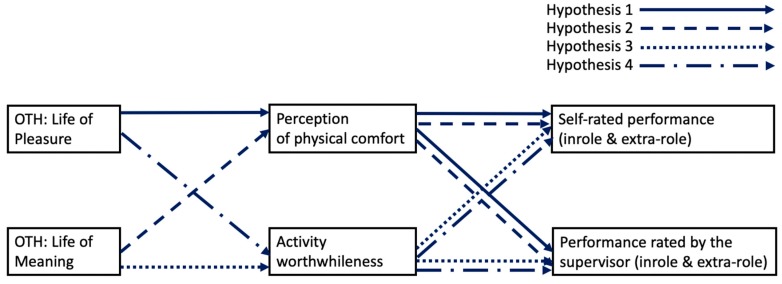
Hypothesized model. Note, OTH = orientations to happiness

Based on the above, the purpose of the present research is to study whether different orientations to happiness are related to hedonic or eudaimonic daily experiences at work, which, in turn, would lead to performance. All the hypothesized relationships are depicted in Figure 1. Paying attention to these associations would help to explain which orientation to happiness and which path (through hedonia or eudaimonia) is the most relevant for performance.

### 1.4. Supervisor Ratings

An additional aspect of the present research is that it takes into account both employees’ ratings of their own state in-role and extra-role performance and their supervisors’ ratings of their overall in-role and extra-role performance during the measurement period. We consider it necessary to combine employees’ ratings of their state performance with the supervisor’s evaluation of their performance in order to avoid employees’ leniency or self-deception in self-ratings [70]. This bias may appear because respondents tend to address not only past behavior, but also their expectations of current and future behavior [71]. By using supervisor evaluations of employees’ performance, we ensure that we are using evaluations that meta-analyses have found to have the highest mean reliability [72].

### 1.5. The Role of Physical Thermal Comfort

Furthermore, given that perceived comfort in offices has been shown to be affected by actual physical thermal comfort (operationalized as predicted mean vote, PMV) [73], and that objective physical office characteristics are linked to the subjective way people perceive them [50], we considered it necessary to control this “hard” physical variable in the study. We believe both considerations are important contributions of the present study.

## 2. Methods

### 2.1. Sample and Procedure

As suggested by Sonnentag [11], research “should strive for more fine-grained assessments by realizing more than one measurement point per day in order to gain more insight into the temporal order of the underlying processes” (p. 284). Therefore, in the present study, we gathered data using short questionnaires (with Likert response scales) that are referred to as “diary study”. A diary design makes it possible to capture respondents’ responses at the moment or shortly after a phenomenon occurs, which is regarded as an appealing method to apply in research on well-being [11] and comfort [74] in office workers.

In the present study, we approached sixty-eight white-collar office employees from five organizations located in the Valencian Region in Spain. These organizations represent various sectors: professional services, mixed sector (2), higher education, public sector (1), furniture industry, private sector (1), and banking, private sector (1). Working conditions in all the organizations satisfy the workplace safety rules conditions established by law.

The workers filled out diary questionnaires that measured perceptions of comfort, activity worthwhileness, and self-rated performance twice a day on four consecutive days (for more details, please see the “Variables and their operationalization” section (Section 2.2)). Because some of the participants were away from the office during part of the work day, we could not collect data at 73 time points. In this way, we obtained 471 data collection points in total. In addition, the participants were approached with a baseline questionnaire about their orientations to happiness (i.e., life of pleasure and life of meaning) about four days prior to the diary measurement week. Finally, each employee’s performance during the measurement week was evaluated by his/her supervisor at the end of the week. We intended to collect data from each participant on the work team simultaneously. However, in some instances, the time of data collection slightly differed due to the limited availability of the employees in their offices. All the data were treated in a confidential way and participation in the study was voluntary. This research received ethical approval from the University Ethics Committee. The characteristics of the study sample are shown in Table 1. The average age was 39.59 years (standard deviation (SD) = 8.62 years).

We recorded sensor physical thermal comfort (predicted mean vote, PMV) data at 5-minute intervals. In order to obtain the indicators for physical thermal comfort, we calculated a mean score for the time range of the PMV data for each person, starting 30 minutes before filling out the diary and ending at the moment each person completed the diary. We considered this interval to adequately represent the current situation in the office, during the time participants took to fill out the diaries.

### 2.2. Variables and Their Operationalization

Orientations to happiness were measured with an 8-item scale adapted from Peterson and colleagues [10]. In order to ensure the brevity of the scale, we kept only the items that best saturated their factors in the exploratory factor analysis carried out by Peterson and colleagues [10]. This scale had two subscales: life of pleasure (5 items) and life of meaning (3 items). Respondents were asked to indicate to what extent they identified with a series of statements using a response scale ranging from 1 (not at all) to 5 (very much). Sample items were “*For me, the good life is the pleasurable life*” for life of pleasure and “*My life has lasting meaning”* for life of meaning. The mean Cronbach’s *a* for the life of pleasure and life of meaning scales were 0.87 and 0.78, respectively.

Perception of comfort was operationalized as lack of appraisal of environmental stressors [18,36], measured on an 11-item scale based on a measure used by Andersson [75]. The person was asked to evaluate the extent to which s/he was being bothered at that moment by several factors in the workspace (sample item: “*temperature too high*”). The response scale ranged from 1 (not at all) to 7 (very much). The mean Cronbach’s *a* for the scale at the eight time points was 0.84.

Activity worthwhileness was measured with a 3-item scale [67]. The respondents were asked to indicate whether they felt the activities they were currently doing were “*…worthwhile and meaningful*” (sample item). The response scale ranged from 1 (not at all) to 7 (very much). The mean Cronbach’s *a* for the scale at the eight time points was 0.80.

Work performance (self-rated by the employee) was measured with 6 items assessing office workers’ in-role and extra-role performance. Sample items were “*I have been performing well*” for in-role performance and “*I have done more than what was expected of me*” for extra-role performance [76,77]. Respondents were asked to evaluate to what extent they agreed with different statements about their current performance, using a response scale ranging from 1 (not at all) to 7 (very much). The mean Cronbach’s *a* for the scale at the eight time points was 0.78. Work performance was treated as a global score.

Work performance (rated by the supervisor) was measured with the same 6-item measure used to assess office workers’ self-rated in-role and extra-role performance [76,77], but with the items adapted so that they could be responded to by the employee’s supervisor. A sample item was *“S/he has voluntarily done more than what was required of him/her”.* The supervisors were asked to evaluate to what extent they agreed with different statements about the recent work performance of each of their employees, using a response scale ranging from 1 (completely disagree) to 7 (completely agree). The mean Cronbach’s *a* for the scale at the eight time points was 0.90.

#### Control Variables

Sex: Because some studies showed that gender is a factor that can influence the individual appraisal of stressors [78,79], we included sex as a control variable in our analyses.

Predicted mean vote (PMV) is a measure of objective physical comfort, and it is a function of several variables: air temperature (*t*_a_ in °C), mean radiant temperature (*t*_mrt_ in °C), relative air velocity (*v* in m/s), air humidity (i.e., vapor pressure, *p*_a_ in kPa), activity level (i.e., metabolic rate, *M* in W/m^2^), and clothing insulation (*I*_cl_ in clo), and it can be expressed by the following formula: PMV = *ƒ*(*t*_a_, *t*_mrt_, *v*, *p*_a_, *M*, *I*_cl_) [73]. PMV represents the mean thermal sensation vote for building occupants on a standard scale, where zero is the desired value, representing thermal neutrality, and the comfort zone is when PMV falls within the recommended limits (−0.5 < PMV < 0.5) [80]. Therefore, we expected that there would be a non-linear, quadratic relationship between PMV and perception of comfort (there would be a perception of lack of comfort on both extremes of the PMV scale). PMV was measured using a BAPPU-evo multi-measuring device for workplace analysis (ELK GmbH Ingenieurbüro für Elektronik).

### 2.3. Analyses

In this study, we use a diary design and a multilevel approach. Because a multilevel approach allows for analyzing states that occur in different time points and for integrating them in a measure for each subject, we consider it to be an adequate approach for our study. The repeated data in this study can be considered as multilevel because repeated measurements of states at Level 1 (*n* = 423) are nested within persons at Level 2 (*n* = 59). Because in this study we are focused on the relationships between constructs at the person level, we assess the relationships at the Level 2 (‘person level’ or between level). This level includes between-person variations. We used MPlus 7.1 software [81] to carry out multilevel structural equation modeling (MSEM) using maximum likelihood estimation with the robust standard errors (MLR) method. MSEM is adequate and recommended for assessing mediation effects in nested data [82]. MSEM decomposes the covariance matrix of the population into two separate covariance matrices (i.e., within-group and between-group) and then tests a model for each level of the nested data [83]. In order to further test the hypotheses about the existence of cross-relationships between orientations to happiness and their non-matching momentary domains, we carried out two alternative models and compared their fit. In the first one, we allowed for paths between non-matching orientations and states, whereas, in the second one, we did not allow for these cross-relationships.

We calculated confidence intervals using the Montecarlo method for assessing mediation (MCMAM) [84] with 20,000 repetitions to test the significance of the indirect effects. The model fit was assessed by examining the RMSEA (root mean square error of approximation), CFI (comparative fit index), TLI (Tucker–Lewis index), and SRMR (standardized root mean square residual) goodness of fit statistics. Acceptable fit between the hypothesized model and the observed data exists when the model fulfills the following criteria: RMSEA < 0.06, CFI and TLI > 0.95, and SRMR < 0.08, whereas an acceptable fit exists for the following values: RMSEA ≤ 0.08, CFI ≥ 0.90, TLI ≥ 0.90, and SRMR ≤ 0.10 [85,86,87,88,89,90].

## 3. Results

Table 2 presents descriptive statistics for the variables of interest. Prior to the MSEM analyses, we calculated the intraclass correlation coefficients (ICCs) for each dependent/mediating variable and we obtained the following information about the proportion of total variance due to variance between individuals: perception of comfort (ICC = 0.87), activity worthwhileness (ICC = 0.69), and performance (ICC = 0.72).

To test the predictive validity of the hypothesized factors at the ‘person level’ of the nested data structure, we used two structural equation models for the multilevel data to predict employees’ performance. The fit indices of the first multilevel SEM, in which we allowed for paths between non-matching orientations and states, showed excellent fit to the data: χ2 (df) = 5.934(7), *p* = 0.55, RMSEA = 0.000, CFI = 1.000, TLI = 1.039, SRMR (within/between) = 0.029/0.027). The alternative model that did not allow for cross-relationships between non-matching orientations and states obtained a poor fit (χ2(df) = 23.468(9), *p* < 0.05, RMSEA = 0.058, CFI = 0.87, TLI = 0.59, SRMR (within/between) = 0.029/0.089), which was substantially worse than the initially hypothesized model allowing for cross-relationships (∆RMSEA = 0.058, ∆CFI = 0.132, ∆TLI = 0.45, ∆SRMR (within/between) = 0.000/0.062).

**Figure 2:**
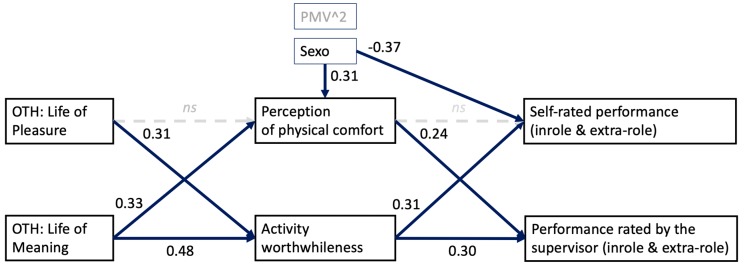
Multilevel structural equation model of the indirect effects of orientation to happiness on work performance at the person level. Note, OTH = orientations to happiness. All relationships in bold are significant at the *p* < 0.05 level.

The results reveal that, at the person level, there is a significant indirect effect of the level of life of pleasure on self-rated performance through activity worthwhileness (95% confidence interval using the Montecarlo simulation (lower limit, LL = 0.001, upper limit, UL = 0.245)), a significant indirect effect of the level of life of meaning on self-rated performance through activity worthwhileness (95% confidence interval using the Montecarlo simulation (lower limit, LL = 0.011, upper limit, UL = 0.429)), a significant indirect effect of the level of life of meaning on performance rated by the supervisor through activity worthwhileness (95% confidence interval using the Montecarlo simulation (lower limit, LL = 0.001, upper limit, UL = 0.398)), and a significant indirect effect of the level of life of meaning on performance rated by the supervisor through perception of comfort (95% confidence interval using the Montecarlo simulation (lower limit, LL = 0.002, upper limit, UL = 0.262)), see Figure 2. In addition, at the person level, there was one indirect significant effect at a 90% confidence level (which is equivalent to a more liberal *p* < 0.10 significance threshold), using the Montecarlo simulation, which was an indirect effect of the level of life of pleasure on performance rated by the supervisor through activity worthwhileness (lower limit, LL = 0.007, upper limit, UL = 0.223). The results support Hypothesis 2, which states that “The relationship between life of meaning and work performance (self-rated and rated by the supervisor) will be mediated by perception of comfort.” Moreover, the results support Hypothesis 3, which states that “The relationship between life of meaning and work performance (self-rated and rated by the supervisor) will be mediated by activity worthwhileness.” Finally, the results yield partial support for Hypothesis 4, which states that “The relationship between life of pleasure and work performance (self-rated and rated by the supervisor) will be mediated by activity worthwhileness.” Hypothesis 1, which states that “The relationship between life of pleasure and work performance (self-rated and rated by the supervisor) will be mediated by perception of comfort” is not supported. The model explained 33% of the variability in self-reported performance (*R*^2^ = 0.333) and 20% of the variability in performance rated by the supervisor (*R*^2^ = 0.196).

## 4. Discussion

The purpose of the present research was to study whether different types of OTH (i.e., life of pleasure and life of meaning) are related to hedonic (i.e., perception of comfort) and eudaimonic (i.e., activity worthwhileness) daily experiences that, in turn, would improve work performance (self-reported by the employee and rated by the supervisor). The results show that, at the person level, there are significant indirect effects of: (a) life of pleasure on self-rated performance through activity worthwhileness, (b) life of meaning on performance (self-rated and rated by the supervisor) through activity worthwhileness, and (c) life of meaning on performance rated by the supervisor through perception of comfort.

These results support the OTH theory [10], showing that well-being can be pursued through two distinct but complementary paths: the hedonic pursuit of pleasure (i.e., life of pleasure) and the eudaimonic pursuit of meaning (i.e., life of meaning) [9]. In addition, the results are consistent with the affective events theory (AET) [13], which postulates that trait-like personal orientations can influence momentary experiences, such as more hedonic (e.g., perception of comfort) or more eudaimonic (e.g., activity worthwhileness) experiences of well-being [14]. Finally, the results support the happy-productive worker thesis, which proposes that “happy” workers will perform better than “less happy” workers [40,41].

The results of the present study expand the empirical evidence obtained in the previous research. In the first place, consistent with Fisher [21] and Basch and Fisher [22], who showed a mediation chain from affective dispositions through affective reactions to work performance (i.e., affective commitment and spontaneous helping behavior) through affective reactions, we show that there is an indirect path from OTH dispositions to work performance through well-being experiences. We contribute to and expand the existing research by showing the mediating role of both hedonic and eudaimonic daily experiences at work on performance.

Accordingly, our results provide support for the assumption that ‘given orientations shape conduct’ [10], and for previous results that show an association between a life of meaning and the eudaimonic experience of activity worthwhileness [15,17] and hedonic experience [15,16]. Furthermore, it agrees with studies that found significant associations between a life of pleasure and experiences of activity worthwhileness [15]. Furthermore, our results resonate with studies showing that both positive hedonic experiences and perception of comfort [40,51,52], as well as eudaimonic experiences of activity worthwhileness [11,20], are associated with work performance. However, our results point out that there is no direct link from the perception of comfort to self-rated performance.

An interesting result, contrary to the one obtained by Grimm and colleagues [15], is that the mediation path from OTH life of pleasure to performance through perception of comfort was not significant. This result suggests that drawing pleasure-oriented employees’ attention to comfort in their offices may not be an efficient way to improve their performance. Instead, their attention should be drawn to the substance and potential meaning that resides in the activities each person carries out at work.

Thus, the results show that there are more significant paths from or through eudaimonia to performance (five paths) than from or through hedonia to performance (three paths). These results suggest that the “paths from or through eudaimonia” are more efficient ways to increase work performance. They suggest that the pursuit and/or experience of eudaimonic happiness is more beneficial for work performance than the pursuit and/or experience of hedonic happiness. These results coincide with Sonnentag [11], who suggested that eudaimonic well-being is an important predictor of performance. This finding adds important information about the antecedents of the experience of the two types of well-being, expanding our knowledge about the complex chains of antecedents of performance.

The results show that there are relationships between matching well-being domains (i.e., life of meaning and activity worthwhileness) and between non-matching domains (i.e., life of meaning and perception of comfort, and life of pleasure and activity worthwhileness). This result might be due to the fact that each activity that one carries out at work might entail a mixture of hedonic and eudaimonic experiences, which is consistent with the ‘blended activities’ concept proposed by Steger and colleagues [91], who suggest that activities can be rated as a combination of both hedonia and eudaimonia, and, therefore, behaviors can be experienced as a blend of pleasure and meaning.

Finally, we might also try to explain the connection between life of meaning and activity worthwhileness by drawing on cognitive dissonance theory [92], because the orientation to meaning can induce pain (or at least it does not have to be pleasant because it does not aim to satisfy pleasures). According to cognitive dissonance theory, employees might be motivated to increase their perception of the worthwhileness of their activities in order to diminish the unpleasant state provoked by cognitive conflict.

### 4.1. Limitations

Some limitations warrant a cautious interpretation of the results of this study. First, the sample in the present study included rather comfortable offices with generally good environmental conditions that did not offer clearly out-of-range conditions. This characteristic might have had an impact on the restriction of range of the perception of comfort, which might be the reason no relationship was found between perception of comfort and self-reported performance. In order to increase the variability in the perception of comfort, future research could expand the sample to investigate offices with indoor environment characteristics that clearly exceed the acceptable ranges. Second, although in the present study we control for objective physical thermal comfort (PMV), which is one of the most important indoor environmental quality (IEQ) conditions [93,94,95,96], we do not include other relevant physical measures of other environmental stressors. Because studying the relationship between physical comfort and the perception of comfort in offices is not the main scope of our study, we suggest that future research could consider measuring other environmental stressors using physical measures. Finally, in the present study, we do not distinguish between primary and secondary appraisals of comfort, and future research could discriminate between these two types of appraisals.

### 4.2. Contributions

The main contribution of this study is the analysis of the relationships between OTH, the experience of both hedonic and eudaimonic well-being at work, and their consequences for performance, shedding light on the dynamic nature of hedonic and eudaimonic facets of well-being and performance, which, until now, had not been sufficiently explored [11]. Therefore, our study follows the advances in the measurement of subjective well-being [65] by distinguishing between day-to-day ‘pleasure’ experiences at work and the ‘worthwhileness’ facet of the activities carried out on a daily basis at work [66,67]. Consequently, the present study adds to the existing research on the OTH [13] and the happy-productive worker thesis [40] by approaching the complexity of the phenomena studied using a diary study design that includes two measurement points per day on four consecutive days. In this way, the present study provides “more fine-grained assessments” to “gain more insight into the temporal order of the underlying processes” [11], instead of assuming that well-being is a global experience that refers to longer time periods [76]. This is especially important in better understanding these constructs as the perception of environmental stressors (i.e., comfort), due to their variability throughout the day, as well as the dynamic nature of eudaimonic well-being [11]. Furthermore, in this study, we combine employees’ ratings of their state performance with the examination of levels of employees’ performance, as evaluated by their direct supervisors, in order to avoid employees’ leniency or self-deception in their self-ratings, which tends to occur in cases of general or trait judgments of performance [70]. Also, in the present study, we test two alternate models that help to establish the veracity of our conceptual model. Finally, we objectively measure physical thermal comfort by using the PMV index in order to control its impact on the perception of comfort in the offices.

### 4.3. Implications

The results of this study suggest that the “paths from or through eudaimonia” are more efficient ways to increase work performance. Knowledge about this discreet ‘triumph of matter (eudaimonia) over form (hedonia)’ and the psychological processes that link OTH to performance is essential in order to implement effective measures that can promote meaningful performance at work. In this regard, this study has practical implications. First, training courses could be proposed to increase the perception of activity worthwhileness. Second, jobs could be designed in such a way that employees would be given more autonomy, more collaborative work on tasks with opportunities for more meaningful tasks, or possibilities for job crafting. This could be especially relevant in new ventures working on both job and office design: this study suggests that it is more important to think about the meaning the activities have for each person than to merely highlight comfort and aesthetics in offices. Finally, there might be implications for the employees themselves: this study is an invitation to turn to their daily activities, analyze them with a positive mindset, and try to find greater meaning in what they do on a daily basis.

## 5. Conclusions

To conclude, the results from this study suggest that, in offices that are within a good comfort range, environmental conditions do not tell the whole story. The extent to which people evaluate their activities at work as valuable is also vital for organizational outcomes such as employee performance. Nowadays, office design and office comfort are considered important aspects of office buildings that promise well-being and economic benefits. However, following Herzberg [97], it could be beneficial to complement the hygiene factor of working in an office that meets good comfort standards with the motivational factor of performing fulfilling and meaningful daily activities at work. Activity worthwhileness might not be as visible at first glance as the office design is, but it can be worthwhile. In this way, we might discover and foster new “eudaimonia paths” that will make us happier and better performers.

## Figures and Tables

**Table 1:** Sample Characteristics.

Characteristics	n (%)
Sex	
Female	43 (63.2)
Male	25 (36.8)
Marital status	
Single	21 (30.9)
Married/Living with partner	46 (67.6)
Separated/Divorced	1 (1.5)
Highest education level reached	
Compulsory education (primary or secondary)	1 (1.5)
Occupational training	11 (16.2)
University degree (Graduated)	16 (23.5)
University degree (MA, Msc)	36 (52.9)
PhD	4 (5.9)
Other	0 (0)
Job level	
Manager	3 (4.4)
Highly-qualified professional	25 (36.8)
Technician	15 (22.1)
Administrative work	20 (29.4)
Auxiliary work	0 (0)
Other	5 (7.4)
Type of contract	
Permanent	57 (83.8)
Temporary	8 (11.8)
Other	3 (4.4)
Salary	
Less than 600€	4 (5.9)
Between 600€ and 1000€	1 (1.5)
Between 1000€ and 1499€	28 (41.2)
Between 1500€ and 1999€	23 (33.8)
Between 2000€ and 3000€	12 (17.6)

Note. *n* = 68; the number in brackets represents the percentage of the total sample.

**Table 2:** Descriptive statistics for the variables of interest.

Variable	*M*	*SD*	Min	Max
Life of pleasure	3.44	0.84	1.00	5.00
Life of meaning	3.51	0.67	1.00	5.00
Perception of comfort	2.01	0.99	1.00	6.60
Activity worthwhileness	5.20	1.21	1.00	7.00
Performance—self-rated by the employee	4.99	1.10	1.67	7.00
Performance—rated by the supervisor	5.70	0.91	2.17	7.00

Note. The descriptive statistics for activity worthwhileness, perception of comfort, and self-rated performance refer to the mean values of these variables over the eight time points during the measurement week. M = mean, SD = standard deviation.
